# The Use of Platelet-Rich Plasma (PRP) for the Management of Non-union Fractures

**DOI:** 10.1007/s11914-020-00643-x

**Published:** 2021-01-04

**Authors:** Christian Andersen, Nicholas M. Wragg, Maryam Shariatzadeh, Samantha Louise Wilson

**Affiliations:** 1 National Centre for Sport and Exercise Medicine, School of Sport, Exercise and Health Sciences, Loughborough University, Epinal Way, Loughborough, Leicestershire LE11 3TU UK; 2 Centre for Biological Engineering, Wolfson School of Mechanical, Electrical and Manufacturing Engineering, Loughborough University, Epinal Way, Loughborough, Leicestershire LE11 3TU UK; 3 School of Pharmacy and Bioengineering, Guy Hilton Research Centre, Thornburrow Drive, Hartshill, Keele University, Stoke-on-Trent, Staffordshire ST4 7QB UK

**Keywords:** Bone healing, Fracture, Non-union, Platelet-rich plasma (PRP)

## Abstract

**Purpose of Review:**

The treatment of non-union fractures represents a significant challenge for orthopaedic surgeons. In recent years, biologic agents have been investigated and utilised to support and improve bone healing. Among these agents, platelet-rich plasma (PRP) is an emerging strategy that is gaining popularity. The aim of this review is to evaluate the current literature regarding the application and clinical effectiveness of PRP injections, specifically for the treatment of non-union fractures.

**Recent Findings:**

The majority of published studies reported that PRP accelerated fracture healing; however, this evidence was predominantly level IV. The lack of randomised, clinical trials (level I–II evidence) is currently hampering the successful clinical translation of PRP as a therapy for non-union fractures. This is despite the positive reports regarding its potential to heal non-union fractures, when used in isolation or in combination with other forms of treatment.

**Summary:**

Future recommendations to facilitate clinical translation and acceptance of PRP as a therapy include the need to investigate the effects of administering higher volumes of PRP (i.e. 5–20 mL) along with the requirement for more prolonged (> 11 months) randomised clinical trials.

## Introduction

The US Food and Drug Administration (FDA) defines a long bone non-union as a fracture that has failed to heal within the first 9 months following injury and has shown no signs of healing for at least 3 consecutive months [1]. However, there is currently no standardised system to define a non-union, so fractures can also be classified as a non-union outside of these parameters (Table 1). The diagnosis of non-union is dependent upon a number of factors including the location of injury, since healing times vary depending upon the type and location of the bone; and weight-bearing bones, such as the femur and tibia, often require the longest healing times to achieve union [2]. A fracture can also be classified by a clinician as a ‘non-union’ when he/she believes the fracture has little or no potential to heal [3]. Specific examples of this arise when there is a persistent gap at the fracture site several months following the initial injury, and/or in instances whereby the patient suffers from persistent pain at the fracture site long after the initial pain of the break has occurred [4].

**Table 1:** Classification of non-union fractures adapted from Panagiotis et al. 2005 [5]

Classification	Description/pathology
Hypertrophic (hypervascular, viable, vital)	Inadequate immobilisation, yet adequate blood supply. In radiographs, callus formation is decreased with an elephant-foot or horseshoe configuration observed.
Atrophic (avascular, non-viable, avital)	Poorly vascularised non-union, resulting in poor potential for forming bone cells and, therefore, delayed healing. There is little callus formation around the fibrous tissue–filled fracture gap.
Oligotrophic	The body can initiate a healing response but is unable to complete the fracture formed between the two ends of the bone.
Septic	A bacterial infection impedes the healing process. Typical bacterial strains that inflict non-union sites include coagulase negative *Staphylococcus* spp. and *Propionibacterium* spp.
Pseudoarthrosis	Persistent motion at fracture site causes formation of false joint (joint that develops at the site of a fracture), often producing synovial fluid.

Impaired healing occurs in 10–15% of fracture patients, which ultimately can lead to a non-union [5]. Characteristics of an impaired healing process include persistent pain, poor bridging of the fracture site by callus formation and a persistent radiolucent line at the fracture which can be visualised via X-ray [6]. Risk factors associated with non-union include increasing age and alcohol consumption, smoking and deficiencies in vitamin D, calcium or protein [4].

Non-union fractures provide a socioeconomic burden that puts pressure on already strained healthcare systems [6]. In the UK, the average cost of long bone non-union treatment is £29,204 per patient; in Canada, 67–97% of the total costs of tibia fractures were specifically related to non-unions in 2014 [7]. Within European healthcare systems, this range narrowed to 82.8–93.3% in the same year [7]. Non-unions are a significant source of morbidity and have a serious impact on patient quality of life (QoL). This is because delayed healing requires additional hospitalisation and the requirement for extensive, often uncomfortable surgical treatments which take, on average, a further 4–8 months to recover from (assuming no further issues arise during treatment and rehabilitation) [7]. The current ‘gold standard’ for treatment of non-union fractures is the use of autologous cancellous bone grafts [8]. Bone from another part of the body is transplanted to the non-union site where it can act as a scaffold on which new bone can grow. Bone grafts provide the non-union site with a fresh source of bone cells to help ‘jump start’ the healing process [4]. However, researchers are currently exploring alternative therapies and treatment options, largely due to the limited supply and donor site morbidity associated with autologous grafts [8]. One potential alternative, which is gaining popularity in the literature, is the use of platelet-rich plasma (PRP).

The aim of this review is to use the current literature to evaluate the effectiveness of PRP in accelerating the healing of non-union fractures. This can be used to inform as to whether PRP may eventually have the potential to become a primary treatment perceptive option.

## Methods

As a part of the systematic literature search, the following inclusion criteria were utilised: clinical reports of any level of evidence, published in the last 20 years (2000–2020) using PRP for the treatment of fractures, with a specific focus on non-unions. Keyword strings of (((‘platelet rich plasma’ OR ‘PRP’))) AND ((‘bone fracture’ OR ‘non-union fracture’ OR ‘delayed healing’)) AND (‘human’ OR ‘clinical’) were used to identify relevant English language articles using the electronic database, PubMed. Scopus, Google Scholar and Springer were also utilised to search for additional resources.

## Results

**Fig. 1:**
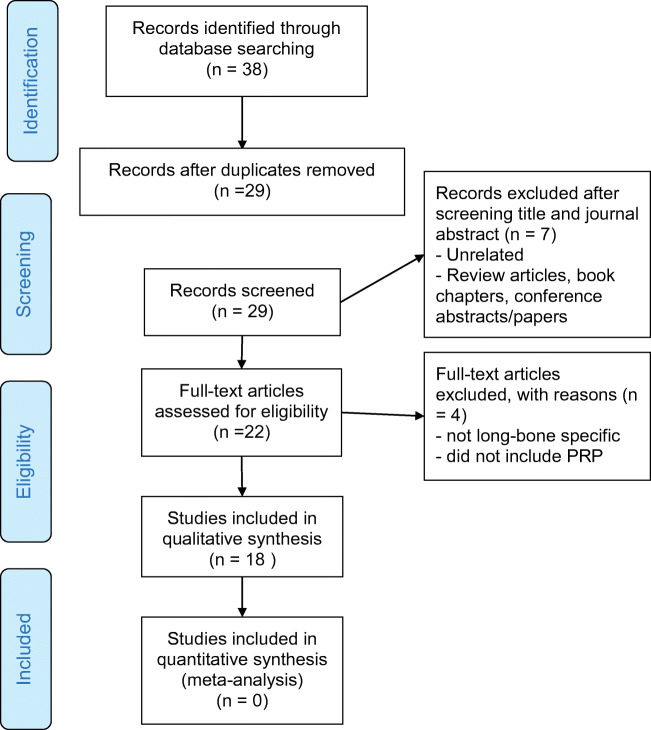
Prisma flowchart of study selection criteria

Using the search terms described returned 38 articles that were eligible for inclusion. The removal of duplicate records resulted in 29 records being screened for relevance (Fig. 1). The titles and abstracts were screened, and 7 articles were removed since they were unrelated and/or review articles, book chapters or conference proceedings. Four articles were removed since they referred to soft tissues. Eighteen studies were included in a qualitative synthesis. Due to the diversity of protocols implemented and the significant variation in outcome measures that were reported, it is extremely difficult to directly quantitatively compare the studies. Thus, a narrative critique was deemed to be most appropriate to review and present the relevant literature. The search revealed that the vast majority of literature has reported benefits of PRP in accelerating bone regeneration. The optimum dose of PRP for treating non-unions is currently undetermined, and the lack of standardisation regarding the preparation and delivery of PRP is retarding clinical translation. Therefore, this review also aims to provide recommendations for successful clinical translation, uptake and acceptance.

### PRP and Its Role in Regeneration

The use of PRP in tissue regeneration is a rapidly evolving area for both clinicians and researchers and is being employed in various fields, including osteoarthritis [9], rotator cuff repair [10, 11] and bone regeneration [12]. This is because autologous platelet concentrations offer an easy, cost-effective method to obtain the high concentrations of specific growth factors including platelet-derived growth factor (PDGF), vascular endothelial growth factor (VEGF), transforming growth factor (TGF beta 1 and 2) and insulin-like growth factor (IGF-1) which are required for tissue healing and regeneration [9]. A healthy individual has a baseline platelet count between 1.5 and 4.5 × 10^5^/μL [12]; to be deemed as therapeutically beneficial, a platelet concentration of 4–5 times that of baseline should be present. The preparation of PRP varies slightly in published literature. However, in general, blood is drawn into a tube which is often treated with an anticoagulant [13]. This is followed by centrifugation and activation of the platelets via a chemical agent [13]. Frequently used activators include calcium chloride [13–19] and bovine [17, 20] or autologous thrombin [15, 21, 22]. Thrombin forms a gel-like substance, from which the PRP can be extracted and directly applied to the patient intravenously [14, 17, 23].

Within platelets are granules, which contain numerous growth factors and cytokines that are important in the early stages of bone repair [12]. On activation, following clotting, these platelets release these growth factors, which play a critical role in the production of proteins required for regenerative processes, such as cellular proliferation, matrix formation, osteoid production and collagen synthesis [12].

### Current Use of PRP in Non-union Fractures

Clinical trials have investigated the effect of PRP on non-union healing alone [14, 21, 24, 25] and in combination with other forms of treatment such as the use of mesenchymal stem cells (MSCs) [26, 27], internal fixation and/or nailing [16, 19, 23, 28–30]. When using PRP in isolation to treat a non-union, the therapeutic benefit is divided; in some instances, PRP has been deemed successful in achieving bony union at the fracture site [24, 25, 31] within 11 months of initial injury or surgery [17]. In addition, PRP has been shown to enhance the healing of non-unions when used in conjunction with other forms of treatment such as the ‘gold standard’ autologous bone graft [32, 33], as well as MSCs [27, 34] and internal fixation [16, 28, 29]. Despite the majority of literature reporting the success of PRP in accelerating healing, it has been found to have less of an effect when compared to other forms of treatment, most importantly the use of bone morphogenetic proteins (BMPs), such as rhBMP-7 [35]. This is most likely due to the low concentration of growth factors which can be extracted with PRP in comparison to BMPs [36].

**Fig. 2:**
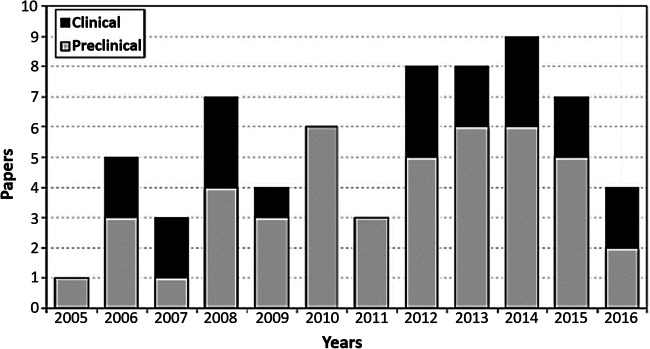
The current proportions of pre-clinical and clinical studies investigating the use of PRP for the treatment of bone defects from 2005 to 2016, reproduced from Roffi et al. (2017) [37] with permission from copyright owner (the authors)

Among the literature concerning the use of PRP specifically for the treatment of non-union fractures, there is currently a lack of prospective randomised clinical trials (RCTs) and, therefore, a minimal amount of literature with level I–III evidence. Instead, studies are predominantly in the form of case series or preliminary studies (level IV evidence) [14–19, 22, 24–27, 29, 31]. The advantage of case series is that they are relatively easy to conduct whilst requiring less time and financial resources in comparison to RCTs, cohort or case-control studies. However, the limitations include a lack of control subjects, which leaves the interpretation of results open to bias. Not only are the majority of published PRP studies case series, but the studies are also predominantly pre-clinical (Fig. 2). Fortunately, the most recent literature [14, 15, 22, 31], summarised by Roffi et al. [37], demonstrated that studies are moving towards a more clinical direction, which is vital for successful translation of any therapy.

### PRP Preparation and Administration

**Fig. 3:**
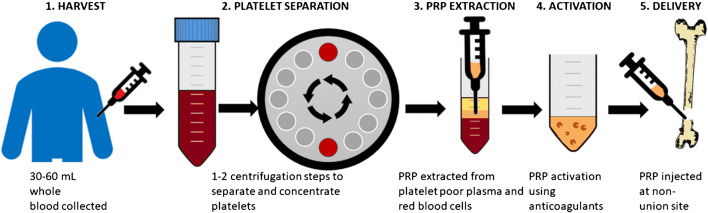
A generalised overview of the PRP process

Despite PRP being widely used for several musculoskeletal pathologies [9–11], there is currently a lack of standardisation with regard to the how PRP is prepared and delivered for treatment of non-union fractures. In general, the PRP preparation process involves the processes of collection, centrifugation to separate out the platelets, extraction of platelet-rich plasma from the red blood cells and platelet poor plasma and activation using an anticoagulant agent (although this is not always the case) followed by administration to the injury site (Fig. 3).

During preparation, the process of centrifugation is utilised for concentrating platelets in all literature. However, the rates of centrifugation differ between studies, ranging from 3200 up to 5200 rpm [15, 19, 21, 30], which leads to different relative forces depending on rotor length, while other studies do not state the rate [7, 35]. Furthermore, centrifugation can potentially lead to the fragmentation of the platelets and the early release of growth factors which will ultimately reduce the bioactivity of the PRP [15]. Thus, activation of the PRP could be influenced by the relative centrifugal forces between studies causing unwanted variation. Therefore, it is recommended that future studies consider ultrafiltration, which potentially offers a more standardised process for PRP extraction [17].

Activation of PRP via the use of chemical anticoagulant agents is also a key component of the preparation process. Throughout the literature, this activator varies, with thrombin, calcium chloride and calcium gluconate being the most common. Thrombin is often combined with calcium chloride (CaCl_2_) [19, 20] or calcium gluconate [15, 21] in an attempt to further increase activation of the platelets. In some literature, details regarding activator use are absent [31], with some studies using none at all [24]. Different reagents have differing half-lives [38], which also has an impact on the duration of the anticoagulant to clear the system. This in turn will have an impact on PRP activation and, therefore, have an impact on the rate of bone regeneration and PRP efficacy. Furthermore, anticoagulant use, particularly those with prolonged half-lives, may limit the suitability of PRP as a therapy for some patient groups including those with anaemia and renal diseases [39]. When analysing PRP delivery methods, it was noted that the volume of PRP delivered varies significantly between studies, with the dose of a single injection varying from 2.5 ml [14] up to 20 ml [24]. The total dose is usually given to a patient as a single dose [21, 24], but may also be divided into multiple injections over consecutive weeks [14, 25] (Table 2). This will likely cause differing levels of efficacy between methods of application as injecting a single dose in comparison to the same dose being divided over several weeks will impact upon the rate of bone regeneration. Dividing a set dose over a period of several weeks will likely delay the intended effect of the injection, slowing the rate of non-union healing. Conversely, the application of multiple equivalent doses of PRP over a prolonged period may increase healing rates, although it is impossible to say for certain since rate of healing is patient specific and RCTs would need to be performed to clarify this.

**Table 2:** Platelet-rich plasma for the treatment and management of non-union fractures: comparison of studies

Author/study	Size and type of non-union	Treatment	Harvest volume	Platelet separation/centrifugation protocol	PRP extraction blood count	Activation/use of anticoagulant	Delivery method/volume	Outcome measure	Comparison/benefit of PRP	Type of study
Bielecki et al. 2008 [17]	Established delayed union and non-union fractures (*n =* 32): Delayed union group (*n =* 12): tibia (*n =* 9), fibula (*n =* 3). Non-union group (*n =* 20): tibia (*n =* 11), fibula (*n* = 1), humorous (*n =* 3), radius (*n =* 2).	Percutaneous injection of autologous platelet-leukocyte-rich gel (PLRG), intervention > 12 months following injury.	For PRP: 108 ml of autologous basilic vein with 12 mL anticoagulant (sodium citrate).	Centrifugation × 1 @ 3200 rpm 12 min.	Preoperative platelet count < 130 × 10^9^/L to meet inclusion criteria. Mean platelet count was 241 ± 64 × 10^9^/L and mean leukocyte count was 7.6 ± 2.57 × 10^9^/L in blood. Platelet counts were increased by 720% and leukocyte counts were increased by 760% on average.	12 mL sodium citrate in 108 mL autologous blood used as anticoagulant for isolation of PLRG. 12 mL PLRP mixed with 3 ml 1600 U/mL bovine thrombin in a 10% calcium chloride (CaCl_2_) solution.	Single percutaneous injection of 15 mL PLRG (12 mL PLRP with 3 mL thrombin solution) directly to site of delayed/non-union under fluoroscopic guidance	Patients followed regularly using clinical examination, roentgenograms, dual-energy X-ray absorption (DEXA) and functional evaluations at days 3 and 3, 5, 8, 12, 18 and 24 weeks	In the delayed union group, the average hospital stay per patient was 1.9 days. Union was observed in all cases. The average time to union was 9.3 weeks (range 5–12 weeks) after PLRG injection. In the non-union group, the average hospital stay was 1.8 days per patient. Union was observed in 13 of 20 cases. The average time to union was 10.3 weeks (range 8–18 weeks) after PLRG injection	Case series
Calori et al. 2008 [35]	Persistent atrophic long bone non-unions (*n =* 120) including tibia (*n =* 15), femur (*n =* 10), humerus (*n =* 15), ulnar (*n =* 12) and radius (*n =* 8) in rhBMP-7 group. Tibia (*n =* 19), femur (*n =* 8), humerus (*n =* 16), ulnar (*n =* 8), radius (*n =* 9) in PRP-treated group.	Comparison of rhBMP-7 mixed with a bio-reabsorbable carrier (3.5 mg Eptoterminalfa, + 1 g collagen, Osigraft, reconstituted with 2–3 mL physiological solution (*n =* 60), compared to PRP (*n =* 60).	54- or 108-mL whole blood in acid acid-citrate-dextrose; 20 mL PRP extracted in total.	Centrifugation 2 × 14 min (no rpm/×*g* information provided)	No values provided.	Acid-citrate-dextrose as an anticoagulant (0.163 mL per 1 mL of blood) used as an anticoagulant.	Local administration during revision surgery. 1× vial rhBMP-7 used in 58/60 patients in rhBMP-7 group, and × 2 vials in remaining two patients (no volumes provided). 20 mL PRP or slightly less delivered to patients assigned to PRP group.	Fracture healing via clinical and radiological evaluation (X-ray and CT scanning where required) performed pre-treatment, followed up at intervals of 1, 3, 6, 9 and 12+ months. Functional outcomes evaluated in terms of presence or absence of pain assessed used a modified VAS.	Rate of union in PRP group (68.3%) significantly lower compared to rhBMP-7 group (86.7%) Pain-free movement was reported by all of 35 patients with upper extremity fractures treated with rhBMP-7 at the 9-month primary end point, compared to 30 out of 33 patients treated with PRP who had a satisfactory functional outcome. The percentage of reported pain with and without weight bearing in patients with lower extremity fractures treated with either rhBMP-7 or PRP was comparable. A lower median clinical and radiographic healing time was observed in the rhBMP-7 group (3.5 months vs. 4 months and 8 months vs. 9 months, respectively)	Prospective RCT
Centeno et al. 2011 [26]	Stable, chronic non-union fractures (*n =* 6) including distal humerus (*n =* 1), sacral base (*n =* 1), 1st metatarsal (*n =* 1), ischial tuberosity (*n =* 1), tibia (*n =* 1), tibia and fibula (*n =* 1).	PRP combined with MSCs, average time of intervention 8.75 months post-fracture (range 4–18 months)	200 mL of heparinised IV venous blood was used to harvest platelet lysate (PL). MSC isolated using 10 mL of marrow blood from right posterior iliac spine (PSIS), and 10 mL from the left PSIS	Centrifugation @ 200×*g* (no time)	~ 30.25 × 10^6^ ± 34.01 MSCs injected into each patient. No PL counts provided.	Heparin used as anticoagulant for isolation of PL (no values); 1000 I.U. heparin/mL used for isolation of MSCs from marrow blood.	Percutaneous injection of autologous bone marrow–derived MSCs and platelet lysate delivered to ‘*several locations*’ at the fracture site under fluoroscopic guidance	Fracture healing via radiographic evaluation (high-resolution X-ray and/or CT imaging) @ 1, 3 and 6+ months	Adequate healing and return to functional recovery seen in 66.7% (4/6) of patients	Case series
Chiang et al. 2007 [20]	Lower extremity long bone atrophic non-union fractures (*n =* 12) of tibia (*n =* 8) and femur (*n =* 4), both septic and aseptic patients treated.	Bone graft enriched with autologous platelet gel (APG). APG sprayed onto the bone graft to form an APG/graft complex which was cut/moulded to bridge the bony defect.	Patients with small defects (*n =* 6) cancellous bone autografts were harvested from the anterior or posterior iliac crest, dependent upon availability. For larger defects (*n =* 6), the graft consisted of cancellous autograft with allograft (3 patients) or Osteoset (3 patients). 45–55 mL autologous intravenous blood used to harvest platelets	Centrifugation @ 3650 rpm, followed by 60 rpm and 3000 rpm (no specific timings provided).	No values provided.	5–7 mL citrate-based anticoagulant used as an anticoagulant for isolation of platelets. PRP and PPP activated using 5000 U purified bovine thrombin ***or*** autologous thrombin and 5 mL of 10% CaCl_2._	Single injection (*n =* 6) or two injections (*n =* 6)	Fracture healing via radiologic evaluation and bone mineral density (BMD) @ 3 days after treatment, followed up at 1, 2, 3, 6 and 12 months. Functional outcomes measured using the short-form 36 health survey (SF-36).	11/12 patients (91.7%) healed on average 19.7 weeks following single treatment, 1/12 patients healed at 21 weeks following a second treatment. All SF-36 sub scores improved, with Significant improvements recorded regarding physical function, role physical, bodily pain, vitality, social functioning and mental health.	Preliminary case study
Duramaz et al. 2018 [19]	PRP group: long bone diaphysis non-unions (*n =* 14); femur (*n =* 8), tibia (*n =* 6) Control group: femur (*n =* 7) and tibia (*n =* 8).	All patients had been previously treated with intramedullary nailing prior to treatment with percutaneous PRP injection into the non-union site under fluoroscopic guidance (*n =* 14) vs. control: exchange intramedullary nailing (EIN, *n =* 15).	55 mL venous blood.	Centrifugation × 3 @ 3650 rpm, followed by 60 rpm and 3000 rpm (no specific timings provided).	No values provided.	500 U bovine thrombin combined with 10% CaCl_2._	Single percutaneous injection of 10 mL PRP plus 1 mL thrombin and 1 mL CaC_l2_ injected into the non-union site under fluoroscopic guidance.	Clinical assessment: Absence of pain/tenderness at site every 2 weeks using VAS. Fracture healing via radiological evaluation every 4 weeks (X-ray and CT scanning performed on all patients) for at least 24 months, mean follow-up 34.97 ± 8.78 months (PRP group) and 33.73 ± 10.53 months (control group).	Mean healing duration was shorter in PRP group compared to controls (16.71 d ± 2.4 weeks vs. 19.07 ± 3.67 weeks), although the difference was not significant. PRP treated patients achieved 92.8% union vs. 80% in the control group. Postoperative VAS scores were reduced in both PRP and control groups, although the results when comparing PRP to controls were not significant.	Retrospective case study, with control group.
Galasso et al. 2008 [16]	Atrophic diaphyseal long bone non-unions (*n =* 22) of tibia (*n =* 11), femur (*n =* 8) and humerus (*n =* 3).	Intramedullary nailing and PRP gel placed in pseudoarthrosis rim during surgery.	50 mL autologous blood, 6 mL PRP extracted.	‘Centrifugation’ (no values/rates).	Preoperative platelet count < 100,000 cells/mL to meet inclusion criteria.	Low-molecular-weight heparin used as anticoagulant (no values). PRP activated with batroxobin and CaCl_2_ (no values) to obtain a gel	6 mL PRP activated with batroxobin and CaCl_2_ (no values) delivered as a gel to the rim of the non-union site.	Clinical assessment: absence of pain/tenderness at site. Fracture healing via radiologic evaluation performed every 45 days until union achieved. Final follow-up at 13 months. Additional CT scans performed if required.	91% (*n =* 20) of patients achieved bony union at 13-month follow-up, average time to union: 2.5 weeks with no infection, complications or limb shortening observed.	Case series
Ghaffarpasand et al. 2016 [30]	Long bone non-union fracture (*n =* 75): femur (*n =* 35), tibia (*n =* 26), humerus (*n =* 11) and ulna (*n =* 3).	Control: saline placebo (*n =* 38) vs. PRP combined with autologous iliac crest bone grafting and intramedullary nailing (femoral factors) or open reduction and internal fixation using steel plates and screws for (humeral fractures) (*n =* 37).	54 mL venous blood from right cubital vein, ~ 5–6 mL PRP extracted	Centrifugation × 1: 3200 rpm 15 min	Preoperative haemoglobin (mg/dL) 13.5 ± 1.8 (PRP), 13.1 ± 2.3 (control); Preoperative platelet count (× 10^6^/μL): 2.1 ± 0.6 (PRP), 2.2 ± 0.8 (control); PRP platelet count (× 10^6^/μL) 9.3 ± 2.6	PRP activated with acid-citrate-dextrose containing gravitational platelet separation system reagent (GPS®) prior to centrifugation followed by 1 g cefazolin and low-molecular-weight heparin following the operation.	5 mL PRP vs. 5 mL saline (placebo group) injected into the periosteum.	Clinical assessment: absence of pain/tenderness at site using VAS. SSracture healing via radiologic evaluation every 45 days for 270 days (9 months). Limb length was also measured and compared with postoperative length and incidence of infection; malunion and non-union were also recorded.	Healing rate in PRP-treated patients 81.1% (placebo 55.3%, *p* = 0.025) with shorter admission (6.6 ± 1.3 vs. 7.7 ± 1.4 days; *p* < 0.001) and healing duration 8.1 ± 1.2 vs. 8.5 ± 10.7 months (*p* = 0.003). No statistical significance in postoperative infection incidence. Malunion was comparable between groups (10.8% vs. 15.8%; *p* = 0.136). Limb shortening lower in PRP group compared to control (*p* = 0.030). Postoperative pain significantly reduced in PRP group at day 45 and 90.	Randomised doubled blinded placebo controlled clinical trial
Golos et al. 2014 [31]	Delayed union of long bones (*n =* 132) including: humerus (*n =* 28), forearm (*n =* 4), femur (*n =* 9) and tibia (*n =* 33).	PRP injection to the fracture cleft on average 4.05 months following diagnosis of delayed union (range 2–5.8 months) with open reduction and plate fixation.	50 mL whole blood (3–4 mL PRP) or 150 mL whole blood (7.8 mL PRP) dependent upon fracture location and cleft size.	No information.	No values provided.	No information.	PRP injection to the fracture cleft under radiographic guidance × 2. Multiple doses dependent upon fracture (predominantly distal tibial and forearm shaft fracture patients), i.e. 3, or 4 injections.	Radiologic evaluation at 6–8 weeks post-treatment followed by subsequent radiographs every 6 weeks until union achieved.	Bone union achieved in in 81.8% of patents (*n =* 108), on average 3.5 months following administration. PRP most successful in healing of forearm fractures (92.8%). Healing rate 82% in tibial fractures. 82.6% in femoral fractures and 66.7% in humeral fractures.	Case series, no controls
Jiang et al. 2016 [25]	Tibia non-union with breakage of the plate (*n =* 1).	Autologous platelet lysate (APL) percutaneous injection ~ 9 months following fracture diagnosis, 6 months post-failed internal fixation surgery.	40 mL peripheral vein blood, ~ 20 mL PRP extracted.	Centrifugation 200×*g* 20 min @ RT; followed by overnight cryopreservation @ − 80 °C, resuscitation at 37 °C and repeated free thaws (‘*more than twice*’); followed by 1.700×*g* for 6 min.	No values provided.	PRP activated with 1000 IU low-molecular-weight heparin sodium followed by 10 mg/mL doxycycline, ratio 1000:1 prior to injection.	5 mL APL injected weekly directly to fracture site under fluoroscopic guidance × 3	Fracture healing via radiologic evaluation at monthly intervals until union achieved	Improved clinical outcome and callous formation at 4- and 6-month post-injection; good bony union at 8 months.	Case study
Labibzadeh et al. 2016 [27]	Aseptic non-union (*n =* 7) of femur (*n =* 4), tibia (*n =* 1), fibula (*n =* 1), tibia and fibula (*n =* 1).	PRP (platelet lysates, PL) combined with bone marrow–derived mesenchymal stem cells (BM-MSCs) MSCs (20–50 million cells per injection). Duration of non-union ranging from 8 months to 11 years.	Umbilical cord blood. BM aspiration: 100–150 mL from iliac crest	Centrifugation 2000×*g* 2 min or 1000×*g* 15 min at 20 °C; followed by 3000×*g* 10 min @ 10 °C; overnight freezing @ − 70 °C followed by thawing at 37 °C and heat inactivation at 56 °C for 30 min. Finally, 900×*g* 30 min.	No values.	No information.	3 mL PL with BM-MSCs implanted into non-union site using fluoroscopic guidance.	Radiological evaluation at 1, 3, 6 and 12 months post-implantation.	57% (4/7) successful bony union with PL + BM-MSCs: @ 2 months (*n =* 1), 6 months (*n =* 2) and 12 months (*n =* 1)	Case series clinical trial, no controls
Mahadik et al. 2018 [29]	Long bone non-union (*n =* 30): femur (*n =* 17), tibia (*n =* 5), humerus (*n =* 5), radius and ulna (*n =* 3).	PRP and internal fixation (open reduction and internal fixation, screwing or nailing dependant on type of fracture following initial failed treatment using conservative/ surgery dependent upon fracture type).	No values.	No information.	No values.	No information.	~ 10 mL injected around fracture site in the periosteum	Clinical assessment: absence of pain/tenderness at site. VAS and radiological evaluation at 1, 2 and 3 months, followed by every 45 days for 12 months. CT scan incorporated if X-ray findings were inconclusive or ambiguous.	All fractures showed radiological evidence within 4 months; 65.25 days in closed fractures and 93.25 days in open fractures. VAS scores reduced from 11 patients with VAS > 6 (severe pain) at 1-month post-intervention to 5 patients at 1 month. At 6 months post-intervention, only 7 patients had mild pain, 23 patients had no pain (VAS = 0).	Prospective case series, no controls
Malhotra et al. 2015 [24]	Established long bone non-union (*n =* 94): tibia (*n =* 35), femur (*n =* 30), humerus (*n =* 11), radius (*n =* 4), ulna (*n =* 12), radius and ulna (*n =* 2). > 90% contact between fracture fragments with suitable open reduction and internal fixation (*n =* 71) or stable reduction with plaster (*n =* 23) immobilisation.	PRP intravenous injection; ~ 9.1 months between injury and PRP intervention (range 7–24 months).	100 mL autologous blood from cubital vein.	‘*Series of centrifugation*’ (no further information provided).	> 2 million platelets/μL.	None used.	15–20 mL dose injected once via intravenous cannula under fluoroscopy guidance (w/o anticoagulant).	Clinical assessment: absence of pain/tenderness at site. Fracture healing via radiologic evaluation @ 1, 2, 3 and 4 months.	87% (*n =* 82) patients achieved union at 4 months, 36% (*n =* 34) showed trabecular bridging via X-ray, 44% (*n =* 41) trabecular bridging at 3 months, 13% (*n =* 12) failed union.	Case series, lack of controls
Mariconda et al. 2008 [15]	Long bone aseptic atrophic non-union (*n* = 20) located in the diaphyseal tract (tibia *n* = 12, humerus *n* = 6), forearm (*n* = 2) compared to historical control group (*n =* 20) with fractures to the tibia (*n* = 12), humerus (*n* = 6) and forearm (*n* = 2).	External fixation using unilateral external fixator supported in compression of long bone non-union supplemented with platelet gel (PG) compared to historical controls Average duration of non-union 311 ± 79 days vs. 333 ± 88 days in control group)	75 mL venous blood yielded ~ 14 mL PRP	Centrifugation × 2: 1200×*g* for 25 min, followed by 1800×*g* 10 min	Counts performed on 5 patients, resulting in ~ 1,075,020 platelets/mL. Mean platelet count concentration 4.1 times greater than the baseline platelet count.	Citrate/citric acid/dextrose used as anticoagulant. Autologous thrombin (0.2 mL/mL of PRP) combined with calcium gluconate (0.2–0.5 mL/mL PPP) used to activate PRP, followed by calcium glutamate (0.2 mL/mL) drop by drop to form the PG prior to injection.	During surgery, PG was percutaneously injected in the interfragmentary space under fluoroscopic guidance.	Clinical assessment: absence of pain/tenderness at site every 2 weeks. Fracture healing via X-ray every month	The study failed to show the clinical usefulness of isolated percutaneous platelet gel supplementation in long bone non-union treated by external fixation. The healing rate of non-union was 90% (18/20) in platelet gel cases and 85% (17/20) in controls, respectively (*p* = 0.633). The mean time until radiographic consolidation in non-union supplemented with platelet gel (147 6 63 days) was not different to the result in the control group (153 6 61 days; *p* = 0.784).	Case series, with retrospective (historical) controls
Sanchez et al. 2009 [13]	Aseptic non-union (*n =* 16): diaphyseal (4 humerus, 4 femur, 4 tibia, *n =* 12), supracondylar (*n =* 4).	Surgical fixation with PRP combined with iliac crest autograft ~ 21 months following prior surgical treatment (*n =* 13); PRP alone (*n =* 3).	65 mL peripheral venous blood.	Centrifugation × 1: 640×*g* 8 min.	No values.	PRP activated with 3.8% (wt/vol) sodium citrate prior to centrifugation followed by 10% (wt/vol) CaCl_2_ prior to mixing with bone graft/administration.	PRP with bone allograft biomaterial injected at exposed injury site in 13 patients (no values), remaining 3 patients were percutaneously injected with 6–8 mL PRP alone every 2 weeks for 6 weeks	Clinical assessment: absence of pain/tenderness at site. Fracture healing via X-ray at 2 weeks followed by ~ monthly intervals until union achieved. CT scan incorporated if no improvement following 10–16 weeks.	Union rate in surgical fixation with PRP combined with surgery = 84.6%, with average union time of 4.9 months.	Dual centre retrospective case series
Say et al. 2013 [14]	Tibia/fibula aseptic delayed union (*n =* 8) and non-union (12) following previous surgery	PRP mean treatment post-injury ~ 6–8 months following fracture surgery.	30 mL peripheral blood from antecubital region	Centrifugation × 1: 1800 rpm 8 min	Platelet count PRP/mL increased by 400% compared to thrombocyte count (no values)	PRP activated with 3.2% CaCl_2_ prior to centrifugation, plus 5.5% CaCl_2_ (50 μL/mL) prior to administration	2.5 mL PRP plus CaCl_2_ activator injected into fracture line under fluoroscopy guidance 3 times over a week	Clinical assessment. Fracture healing via radiologic evaluation at ~ 15 weeks	Delayed union patients: union in 60%; non-union patients: PRP unsuccessful in achieving union when used in isolation following 12-month follow-up	Prospective case series
Tarallo et al. 2012 [22]	Non-traumatic ulna non-union between 1 and 5 cm	PRP combined with iliac crest autograft and compression plate, mean treatment post-injury = 11.5 months (*n =* 10). No control.	450 mL venous blood collected.	Centrifugation × 2: 180×*g* 20 min followed by 580×*g* 15 min.	No values	PRP activated with citrate/citric acid/dextrose (no values) prior to centrifugation, plus autologous thrombin and calcium gluconate prior to administration.	Injection at exposed injury site (no values).	Clinical assessment: absence of pain/tenderness at site. Fracture healing via radiologic evaluation at ~ 21 months (range 7–14 months). VAS and disability assessment for shoulder and hand (DASH).	Union achieved in 90% cases with average union time of 4.11 months. Mean VAS score = 1 at rest, VAS = 2 during activity. DASH = 17	Single-centre prospective study
Tawfik et al. 2017 [21]	Nonhypertonic long bone non-unions with 90% contact between fracture fragments	PRP alone, mean delivery 8 months post-injury. (*n =* 20) No control. 5 patients received additional injection following 6 weeks.	450 mL whole blood collected, ~ 10% used for PRP	Centrifugation × 2: 750×*g* 7 min followed by 5300×*g* 10 min.	Leukocytes 0.15 ± 0.02 × 10^3^/μL. Platelet (whole blood) 251 ± 39 × 10^3^/μL. Platelets (PRP) 989 ± 265 = 10^3^/μL.	~ 20 mL PRP activated with 5 mL autologous thrombin combined with 2 mL calcium gluconate prior to administration	25–30 mL PRP plus activator (thrombin/calcium gluconate) percutaneously injected	Clinical assessment: absence of pain/tenderness at site. Fracture healing rate at 6 weeks via radiologic evaluation followed every 4 weeks until union. Additional CT scans performed at 10–16 weeks if suboptimal outcomes detected.	85% successful union among patients receiving PRP at 17 wks. 75% ‘fair’ results following single injection	Single-centre prospective study
Zhao et al. 2017 [28]	Atrophic non-union of femoral shaft	Control (conventional internal fixation surgery with autologous/allogeneic or artificial bone graft *n =* 46) vs. PRP and conventional surgery (*n =* 46)	30 mL collected from elbow vein.	Centrifugation × 2: 200×*g* 10 min	No values.	PRP (no values) activated with sodium citrate (no values provided) prior to centrifugation plus thrombin prior to administration (100 U/mL)	5 mL PRP plus activator (thrombin) directly injected at exposed injury site	Clinical assessment: absence of pain/tenderness at site. Fracture healing rate at 9 months via radiologic evaluation; VAS at 3, 6, 9 and 12 months	PRP with internal fixation significantly increased healing rate (94% vs. 78% compared to control). Reduced healing time (91.6 days vs. 115.2 days in control). No significant difference in VAS.	Randomised, controlled clinical trial

*APG*, autologous platelet gel; *APL*, autologous platelet lysate; *BM-MSCs*, bone marrow–derived mesenchymal stem cells; *CaCl*_*2*_, calcium chloride; *CT*, computerized tomography; *DASH*, disability assessment for shoulder and hand; *EIN*, exchange intramedullary nailing; *MSCs*, mesenchymal stem cells; *PG*, platelet gel; *PLRG*, platelet-leukocyte-rich gel; *PRP*, platelet-rich plasma; *PSIS*, posterior iliac spine; *rhBMP-7*, recombinant human bone morphogenetic protein-7; *RT*, room temperature; *SF-36*, short-form 36 health survey; *VAS*, visual analogue score

### The Impact of PRP Activation on Bone Regeneration

The majority of published studies currently fail to report key aspects such as platelet concentrations, leukocyte components and activation modalities [37]. This is despite Chen et al. demonstrated how a medium concentration of PRP (2.65 ± 0.2 × 10^9^/mL) induces oestrogenic differentiation of bone marrow stem cells (BMSCs/BM-MSCs), improving fracture healing, whereas a high concentrations of PRP (8.21 ± 0.4 × 10^9^/mL) can inhibit osteogenic differentiation and delay callus remodelling [40]. Labibzadeh et al. reported that leukocyte-rich PRP induced higher proliferation of BMSCs [27], while other literatures have found activation modality to influence the molecules released by the PRP [41]. This highlights that differing concentrations and ultimately levels of activation will affect the efficacy of the therapy.

### The Use of PRP in Isolation to Treat Non-union Fractures

Once PRP has been prepared, treated with an anticoagulant and activated, it is often then applied alone as a form of treatment. Only one study reported PRP to be unsuccessful in achieving union when used in isolation [14]. The study comprised 20 patients, 12 of which were diagnosed with a non-union fracture. These subjects were treated weekly for 6 months [14]. No patient achieved union up to 10 months following treatment. Therefore, the authors concluded that PRP was ineffective in treating non-unions [14]. However, the administration of the PRP was a limitation of the study. A total of 2.5 mL of PRP activated by calcium chloride was injected each week for 3 weeks. A 2.5 mL dose of PRP is relatively small in comparison to other studies; however, it is impossible to determine how the harvest blood and/or delivery volume used in any study compares to the number of platelets and/or leukocytes isolated, since this value is very rarely stated in published studies (Table 2). Furthermore, the size of non-union is widely varying when comparing patients within study groups and separate studies to each other. Moreover, the size/volume of the non-union is not always prescribed in published studies. PRP has been a reported success with a dose of at least 5 mL per injection [28, 30], often applied on at least three separate occasions [25, 31] In addition to this, the PRP was activated using calcium chloride. In the majority of published literature where PRP has successfully induced union, bovine/autologous thrombin was utilised in the activation process [15, 17–19, 21, 22, 28], thus implying that activation using calcium chloride alone and the lower dose of PRP could potentially be key factors in the unsuccessful union of these patients’ fractures.

Despite its common use, bovine thrombin as an activator has been questioned in clinical application, since disease transmission, possible carcinogenesis, availability and cost are all issues related to bovine thrombin [20]. Furthermore, Malhotra claimed that sufficient thrombin is produced via local trauma when the fracture site is infiltrated with a needle [24]. To test this hypothesis, a high volume of 20 mL of PRP was injected on one occasion without thrombin and with a high concentration of platelets (approximately 5 times normal values) [24]. Eighty-seven percent of patients achieved union at the end of the 4-month follow-up period. All of which had an average time of 9.1 months between the injury and the PRP injection [24].

In 2008, Bielecki et al. concluded that PRP is a sufficient method to achieve union as long as the treatment occurs ≤ 11 months following initial surgery [17]. In Malhotra et al.’s study, the 13% of patients who had previously not achieved union were treated with PRP over 12 months following initial diagnosis. However, following the conclusions of Bielecki et al., due to the delay in PRP treatment from diagnosis, the fracture gap most likely became too large for the PRP to have a regenerative effect. Limitations that were acknowledged in the study were that it was not a RCT and only involved non-unions which had more than 90% contact between the fracture fragments. Therefore, findings from this study may not be applicable to more severe non-unions. A more recent study by Tawfik et al. had similar inclusion criteria (more than 90% contact between the fracture fragments) and dose of PRP (20 mL) and found very similar rates (85%) of union among patients [21] in comparison to Malhotra et al. (87%).

### PRP as a Hybrid Treatment

PRP can also be utilised in combination with other forms of non-union fracture treatment, including autografts, compression plates and/or fixation devices [13, 15, 19, 27, 28, 30, 31]. Some studies have investigated the effect of combining PRP with an iliac crest autograft and concluded that the addition of PRP has the potential to enhance healing [13, 22, 30]. However, in these studies, the authors could not attribute the healing of the fracture to the addition of PRP in isolation, due to the lack of randomised control groups. Therefore, no information could be provided regarding the efficacy of one particular method. This is apparent in the case of Tarallo et al. where patients were treated using a bone graft, dynamic compression plate and PRP [22]. Despite this, union was achieved in 90% of cases with an average time to union of 4 months. Ghaffarpasand et al. conducted the only randomised double blinded placebo-controlled clinical trial investigating the effect of PRP on the rate of healing of non-union fractures treated with an autologous bone graft and internal fixation. Patients received either 5 mL of PRP or 5 mL of saline (placebo) during surgery. The healing rate was significantly higher in the PRP group in comparison to that in the control (81.1% vs. 55.3%, *p* = 0.025) [30].

Reports on the effectiveness of PRP and autograft fixation as a non-union fracture treatment are divided. Case series findings have largely been positive in terms of improved rates of non-union healing [13, 17, 20, 26]. Though, the rate of healing cannot be attributed to the use of PRP due to a lack of effective controls. However, in a unique case series by Mariconda et al., the healing rate of union of patients treated with PRP and external fixation (90%) was compared to the healing rate of union of a historical control group (85%), with no significant clinical usefulness of PRP being reported [15]. However, caution should be taken when interpreting this result, since the sample size was relatively small (*n* = 20), limiting the statistical power of the data. A recent randomised, controlled clinical trial regarding the combined use of PRP and internal fixation [28] reported that the addition of PRP to internal fixation significantly increased the rate of healing (94% vs. 78%, *p* < 0.05) whilst reducing healing time (91.6 ± 6.9 vs. 115.2 ± 8.4 days, *p* < 0.05) in comparison to a control group [28]. The study had not only the primary measure of healing rate but also secondary outcome measures assessed using a visual analogue scale (VAS). The VAS was used to measure subjective characteristics that cannot be directly measured such as pain intensity, treatment costs and adverse reactions; all of which are important aspects in a method of treatment [28], particularly when considering clinical acceptance and translation. Since PRP elevated the healing rate, this shortened the treatment time whilst reducing pain and costs.

Two case studies have been conducted investigating if the combination of PRP and MSCs can facilitate healing of non-union fractures, both reporting success with the method [27, 34]. Labibzadeh et al. stated that, when combined with MSCs, PRP is successful in patients who had previously failed to achieve union using the ‘gold standard’ iliac bone graft. This highlights how effective PRP could be in improving bone regeneration in difficult cases of non-union [27]. However, Centeno et al. did reveal a limitation of the method, including the fact that the MSCs isolated from bone marrow aspirate obtained from the iliac crest required a second invasive surgical intervention. This increased the potential risk of infection and causes further pain to the patient [34]. Moving forward, double blinded, controlled trials are still required to assess the clinical efficacy of this treatment.

### The Use of PRP vs. Alternative Treatments

In a well-documented, prospective RCT, it was suggested that rhBMP-7 was a superior bone-stimulating agent compared to PRP. Rather than being injected into the site of the non-union, both PRP and rhBMP-7 were applied locally during revision surgery allowing both methods to be directly compared. Subjects (*n* = 120) in the PRP group had an average non-union duration of 19.2 ± 2.86 months [35]. As discussed above, Bielecki et al. proposed that, for PRP to be a success, the treatment should be made within 11 months of injury or initial surgery [17]. This would suggest that, whilst the rate of union in the PRP group was significantly lower (68.3% PRP vs. 86.7% rhBMP-7), the findings of this study should be interpreted with caution. The authors concluded that administering PRP alone without a bone graft could be deemed non-ideal in terms of exploiting PRP’s innate bone regeneration enhancement capabilities [35]. Since the study, PRP has demonstrated success alone in accelerating rate of union [7, 21, 29], with the majority of the literature investigating the potential of PRP to enhance current treatment strategies [20, 28, 30]. Thus, it is unlikely that PRP as a therapy will be used alone on a large scale. However, PRP could potentially be applied as a minimally invasive method as a way of saving resources in medical care, with no significant difference being found between PRP and intramedullary nailing in achieving union [19]. Larger, randomised controlled studies under optimal treatment conditions are needed to give more powerful and accurate results.

## Conclusion

The consensus from the literature is that PRP is effective in accelerating the healing of non-union fractures. The success it has demonstrated when used in isolation ranges in doses from 2.5 to 20 mL, implying that it could potentially become a primary form of treatment. However, the non-union would have to be inherently stable and PRP would ideally be administered within 11 months of injury or initial surgery, although further investigation is needed to confirm this barrier. Moving forward, it is recommended that RCTs should focus on the effect of injections of at least 5 mL of PRP, since lower doses have not been reported as successful in inducing bone regeneration [14]. The study by Malhotra et al., a major contribution to the research, suggested that the common activator bovine thrombin may not be a necessity [24] and this should be investigated further in future studies to determine whether sufficient thrombin is produced via local trauma when the fracture site is infiltrated with a needle in order to activate the platelets. Other major contributions include the work by Ghaffarpasand et al. and Zhao et al., whereby they have demonstrated the success of PRP and internal fixation as a combination treatment [28, 30]. However, more RCTS are needed to determine if PRP is a more effective bone-stimulating agent for augmenting union in comparison to the likes of MSCs and BMPs. Finally, the most effective method of PRP preparation (rate of centrifugation/ultrafiltration, activator, dose, etc.) and administration (single or multiple injections) needs to be systematically investigated and standardised in order for the therapy to develop further.
